# The six most essential questions in psychiatric diagnosis: A pluralogue part 2: Issues of conservatism and pragmatism in psychiatric diagnosis

**DOI:** 10.1186/1747-5341-7-8

**Published:** 2012-07-05

**Authors:** James Phillips, Allen Frances, Michael A Cerullo, John Chardavoyne, Hannah S Decker, Michael B First, Nassir Ghaemi, Gary Greenberg, Andrew C Hinderliter, Warren A Kinghorn, Steven G LoBello, Elliott B Martin, Aaron L Mishara, Joel Paris, Joseph M Pierre, Ronald W Pies, Harold A Pincus, Douglas Porter, Claire Pouncey, Michael A Schwartz, Thomas Szasz, Jerome C Wakefield, G Scott Waterman, Owen Whooley, Peter Zachar

**Affiliations:** 1 Department of Psychiatry, Yale School of Medicine, 300 George St., Suite 901, New Haven,, CT, 06511, USA; 2 Department of Psychiatry and Behavioral Sciences, Duke University Medical Center, 508 Fulton St., Durham, NC, 27710, USA; 3 Department of Psychiatry and Behavioral Neuroscience, University of Cincinnati College of Medicine, 260 Stetson Street, Suite 3200, Cincinnati, OH, 45219, USA; 4 Department of History, University of Houston, 524 Agnes Arnold, Houston, 77204, USA; 5 Department of Psychiatry, Division of Clinical Phenomenology, New York State Psychiatric Institute, Columbia University College of Physicians and Surgeons, 1051 Riverside Drive, New York, NY, 10032, USA; 6 Department of Psychiatry, Tufts Medical Center, 800 Washington Street, Boston, MA, 02111, USA; 7 Human Relations Counseling Service, 400 Bayonet Street Suite #202, New London, CT, 06320, USA; 8 Department of Linguistics, University of Illinois, Urbana-Champaign 4080 Foreign Languages Building, 707 S Mathews Ave, Urbana, IL, 61801, USA; 9 Duke Divinity School, Box 90968, Durham, NC, 27708, USA; 10 Department of Psychology, Auburn University Montgomery, 7061 Senators Drive, Montgomery, AL, 36117, USA; 11 Department of Clinical Psychology, The Chicago School of Professional Psychology, 325 North Wells Street, Chicago, IL, 60654, USA; 12 Institute of Community and Family Psychiatry, SMBD-Jewish General Hospital, Department of Psychiatry, McGill University, 4333 cote Ste. Catherine, Montreal, QC, H3T1E4, Canada; 13 Department of Psychiatry and Biobehavioral Sciences, David Geffen School of Medicine at UCLA, 760 Westwood Plaza, Los Angeles, CA, 90095, USA; 14 VA West Los Angeles Healthcare Center, 11301 Wilshire Blvd, Los Angeles, CA, 90073, USA; 15 Department of Psychiatry, SUNY Upstate Medical University, 750 East Adams St., #343CWB, Syracuse, NY, 13210, USA; 16 Irving Institute for Clinical and Translational Research, Columbia University Medical Center, 630 West 168th Street, New York, NY, 10032, USA; 17 New York Presbyterian Hospital, 1051 Riverside Drive, Unit 09, New York, NY, 10032, USA; 18 Rand Corporation, 1776 Main St, Santa Monica, CA, 90401, USA; 19 Central City Behavioral Health Center, 2221 Philip Street, New Orleans, LA, 70113, USA; 20 Center for Bioethics, University of Pennsylvania, 3401 Market Street, Suite 320, Philadelphia, PA, 19104, USA; 21 Department of Psychiatry, Texas A&M Health Science Center - College of Medicine, 4110 Guadalupe Street, Austin, TX 78751, USA; 22 Silver School of Social Work, New York University, 1 Washington Square North, New York, NY, 10003, USA; 23 Department of Psychiatry, NYU Langone Medical Center, 550 First Ave, New York, NY, 10016, USA; 24 Department of Psychiatry, University of Vermont College of Medicine, 89 Beaumont Avenue, Given Courtyard N104, Burlington, VT, 05405, USA; 25 Institute for Health, Health Care Policy, and Aging Research, Rutgers, the State University of New Jersey, 112 Paterson St., New Brunswick, NJ, 08901, USA

## Abstract

In face of the multiple controversies surrounding the DSM process in general and the development of DSM-5 in particular, we have organized a discussion around what we consider six essential questions in further work on the DSM. The six questions involve: 1) the nature of a mental disorder; 2) the definition of mental disorder; 3) the issue of whether, in the current state of psychiatric science, DSM-5 should assume a cautious, conservative posture or an assertive, transformative posture; 4) the role of pragmatic considerations in the construction of DSM-5; 5) the issue of utility of the DSM – whether DSM-III and IV have been designed more for clinicians or researchers, and how this conflict should be dealt with in the new manual; and 6) the possibility and advisability, given all the problems with DSM-III and IV, of designing a different diagnostic system. Part I of this article took up the first two questions. Part II will take up the second two questions. Question 3 deals with the question as to whether DSM-V should assume a conservative or assertive posture in making changes from DSM-IV. That question in turn breaks down into discussion of diagnoses that depend on, and aim toward, empirical, scientific validation, and diagnoses that are more value-laden and less amenable to scientific validation. Question 4 takes up the role of pragmatic consideration in a psychiatric nosology, whether the purely empirical considerations need to be tempered by considerations of practical consequence. As in Part 1 of this article, the general introduction, as well as the introductions and conclusions for the specific questions, are written by James Phillips, and the responses to commentaries are written by Allen Frances.

## General introduction

For the full text of the General Introduction to the entire article, the reader is referred to Part 1 [1]. The General Introduction reviewed the history of the article, which originated in a controversy initiated by Robert Spitzer and Allen Frances, Chairmen respectively of the DSM-III and DSM-IV Task Forces, over the ongoing work of the DSM-5 Task Force and Work Groups. In a series of articles and blog postings in *Psychiatric Times,* Frances (at times with Spitzer) carried out a sustained critique of the DSM-5 work in which he focused both on issues of transparency and issues of process and content [2-15].

In the course of this debate over DSM-5 I proposed to Allen in early 2010 that we use the pages of the Bulletin of the Association for the Advancement of Philosophy and Psychiatry (of which I am Editor) to expand and bring more voices into the discussion. This led to two issues of the Bulletin in2010 devoted to conceptual issues in DSM-5 [16,17]. (Vol 17, No 1 of the AAPP Bulletin will be referred to as Bulletin 1, and Vol 17, No 2 will be referred to as Bulletin 2. Both are available at http://alien.dowling.edu/~cperring/aapp/bulletin.htm.) Interest in this topic is reflected in the fact that the second Bulletin issue, with commentaries on Frances’ extended response in the first issue, and his responses to the commentaries, reached over 70,000 words.

Also in 2010, as Frances continued his critique through blog postings in *Psychiatric Times*, John Sadler and I began a series of regular, DSM-5 conceptual issues blogs in the same journal [18-31].

With the success of the Bulletin symposium, we approached the editor of PEHM, James Giordano, about using the pages of PEHM to continue the DSM-5 discussion under a different format, and with the goal of reaching a broader audience. The new format would be a series of “essential questions” for DSM-5, commentaries by a series of individuals (some of them commentators from the Bulletin issues, others making a first appearance in this article), and responses to the commentaries by Frances. Such is the origin of this article. (The general introduction, individual introductions, and conclusion are written by this author (JP), the responses by Allen Frances.

For this exercise we have distilled the wide-ranging discussions from the Bulletin issues into six questions: 1) the nature of a mental disorder; 2) the definition of mental disorder; 3) the issue of whether, in the current state of psychiatric science, DSM-5 should assume a cautious, conservative posture or an assertive, transformative posture; 4) the role of pragmatic considerations in the construction of DSM-5; 5) the issue of utility of the DSM – whether DSM-III and IV have been designed more for clinicians or researchers, and how this conflict should be dealt with in the new manual; and 6) the possibility and advisability, given all the problems with DSM-III and IV, of designing a different diagnostic system. Part 1 [1] of this article covered the first two questions. This text, Part 2, covers the third and fourth questions.

### Question #3: What are the benefits and risks of conservatism?

Given the state of the science of psychiatric disorders, should we design DSM-5 in a conservative manner, with minimal change, or do the state of psychiatric science and the problems in DSM-IV dictate major change?

## Introduction

By way of introducing this question, I will underline two points that play a role in the discussion. The first involves the validity status of the DSM-IV diagnoses; the second involves the role of values in DSM-IV.

Regarding the first point, virtually all discussants – in this article, in the Bulletin discussions [16,17], and in the article by Regier [32] and colleagues cited in the general introduction – agree on a number of conclusions about the DSMs: that the DSM-IV categorical profiles often do not adequately reflect the heterogeneity of presentation in individuals grouped under a particular category, that the operationally defined categories, while achieving the reliability that was their goal and in that way facilitating research across different settings, also inhibit research [33] by constricting it to the boundaries of the diagnostic criteria, that the diagnostic constructs create a high rate of comorbidity, as well as a high rate of NOS diagnoses, that most of the diagnoses fail the test of the original Robins and Guze [34], as well as the additional Kendler, [35] validators, that current findings in genetics and neuroscience do not match up with the DSM-IV categories, and finally that the research findings of molecular genetics and neuroscience regarding psychopathology are at this point quite unsettled.

Given this agreement on such a large number of issues about DSM-IV, it is striking that different commentators draw dramatically different conclusions from them: one group arguing that the problems of DSM-IV and the current state of psychiatric science lead us to a conservative recommendation of minimal change in DSM-5; the other group arguing that the same set of circumstances leads us to a non-conservative recommendation of maximal change. As we know, Allen Frances argued in blogs, articles, and in his Bulletin 1 Philosophyland piece, for the conservative approach. Commentators in the Bulletin discussions argued for or against him: Piasecki and Antonuccio (Bulletin 2,p. 15) and Phillips (Bulletin 1, p. 10) taking the conservative position, and Ghaemi (Bulletin 1, p. 3 & Bulletin 2, p. 33), Waterman and Curley (Bulletin 1, p. 19), and Waterman (Bulletin 2, p. 29) taking an activist stance. That debate continues in the commentaries below.

Regarding the debate, let me refer to a recent piece by Freeman Dyson in the *New York Review of Books*. Writing about the scientific process, Dyson says:

The public has a distorted view of science, because children are taught in school that science is a collection of firmly established truths. In fact, science is not a collection of truth. It is a continuing exploration of mysteries. Wherever we go exploring in the world around us, we fine mysteries. Our planet is covered by continents and oceans whose origin we cannot explain. Our atmosphere is constantly stirred by poorly understood disturbances that we call weather and climate. The visible matter in the universe is outweighed by a much larger quantity of dark invisible matter that we do not understand at all. The origin of life is a total mystery, and so is the existence of human consciousness. We have no clear idea how the electrical discharges occurring in nerve cells in our brains are connected with our feelings and desires and actionsScience is the some total of a great multitude of mysteries. It is an unending argument between a great multitude of voices. It resembles Wikipedia much more than it resembles the *Encyclopaedia Britannica*[36].

If, with Dyson, one’s attitude toward science is that it “is not a collection of truths” but rather “a continuing exploration of mysteries.”, that might lead one toward the activist stance, arguing that we needn’t wait for final truth from genetics and neuroscience to make changes in our nosology, but rather see the latter as an ongoing, dynamic process. If we assume this latter approach, the questions become the practical ones of how to redesign the manual in a way that allows for our tentative advances in scientific understanding without creating more practical encumbrance than benefit.

The counter argument is articulated by Frances and others: that provisional changes made on the basis of genuine, but provisional, scientific findings may well create more harm than good, and that when dealing with suffering human beings, we had better be pretty sure of our “science” – and of its effects – before making changes that will affect how we deliver treatment. The stakes are high in this discussion, and they will carry over into question #4, where the issue of consequence enters the discussion.

The second point with which I introduce this question involves the normative dimension of DSM-IV. In the matter of values, questions regarding change or non-change in the new manual are completely different from those just discussed under the heading of conservatism and validity. In that discussion the leading question was: given that DSM-IV categories don’t achieve real scientific validity, and that the proposed changes won’t accomplish such validity either, are the proposed changes warranted for whatever they will accomplish in inching forward toward scientific validity, or will they create more chaos and harm than benefit?

In the question of values, on the other hand, we face a completely different kind of question: if there are diagnostic categories in DSM-IV that primarily represent value judgments, and whose validity will not be settled by any science of the future, is there any reason not to decide on them now? The obvious candidates for this discussion are, on the one hand, the paraphilias, and on the other hand behavioral disorders like Conduct Disorder and Antisocial Personality Disorder, whose status as valid psychiatric disorders may not lend itself to a scientific answer. We certainly have an awkward model for deciding this kind of question in the instance of homosexuality, whose removal from the DSM was decided by a membership vote in 1973. That vote was an acknowledgment that the question was not to be decided by scientific evidence.

### Commentary: psychiatric nosology: what are the stakes?

G. Scott Waterman, M.D.

University of Vermont Department of Psychiatry

The stakes for everything seem higher these days than ever before. A credit crisis in a small country reverberates through the world economy at the speed of photons. Publication of political cartoons some find offensive leads to deaths many miles away. Formulation by Medicare officials of medical documentation and billing standards changes the practice of medicine and causes the birth of entirely new industries to comply with new regulations. And so it is with psychiatry: “officialness” has never been so official, magnifying the shortcomings of our diagnostic system manifold and emphasizing the importance of seizing opportunities to learn from the past and improve prospects for the future.

It is a cliché that “the people” tend to be more insightful than their leaders. In the case of the DSM diagnostic system, while representatives of official psychiatry debate the wisdom of a variety of potential alterations, the rank and file have in significant ways already passed judgment. In clinical settings it is widely acknowledged that the phenotypes with which actual patients present bear only mild resemblance to those that define individual DSM entities. That ubiquitous observation leads to what has become very familiar and likely destructive cynicism among clinicians, clinician educators, students, and residents about the diagnostic enterprise. In research settings the unsuitability of the DSM nosology for some of the most promising domains of investigation – including genetics, epigenetics, and functional neuroimaging – has long been understood, leading to an inevitably growing disjunction between what is being learned about the etiopathogenesis of psychopathology on the one hand and official taxonomy of psychopathology on the other.

To the extent that the current approach to psychiatric nosology is simply dying of its own flaws, the stakes involved in reformulating it might not seem so high. But despite its weaknesses, its influence is strong and persistent in (among other contexts) psychiatric education and training – precisely those venues to which we must look for hope for the future of the discipline. Judging from the time devoted to it and the examination questions asked about it, imparting to students and residents the algorithms by which DSM diagnoses are assigned is a major curricular commitment. Among the many problems associated with that commitment is an essentialist assumption about the nature of diagnostic categories which, when juxtaposed with the failure of the taxonomy either to describe or to explain phenotypes adequately, results in the cynicism noted above. Other fallacies imparted to our trainees through the vehicle of the DSM are dualist conceptualizations that spuriously distinguish ‘mental’ from ‘physical’ (or, in the language of the DSM, ‘general medical’) illnesses, and the associated but more subtle dichotomous thinking that results in familiar differential diagnostic formulations of the following type:

Major depressive disorder, recurrent, severe with psychotic features

Rule-out bipolar disorder, most recent episode depressed, severe with psychotic features

Rule-out substance-induced mood disorder with depressive features

Rule-out mood disorder due to a general medical condition

Leaving aside the capital offenses committed against the conventions of English syntax, the following questions must be considered: 1) Is it likely that severe recurrent psychotic depression, even among patients with no discernable histories of mania, shares more important features with bipolar disorder than with unipolar depression? How does expressing differential diagnosis in this way facilitate incorporation of forthcoming evidence on this question? 2) How, exactly, does one “rule-out” ***any*** of these entities? 3) Is it likely that substance use or “general medical conditions” are themselves sufficient causes of (in this case) full depressive syndromes, and thus represent clinical entities distinct from primary or idiopathic mood disorders? Is it more likely that inheritance of various alleles in whose presence events in the external (i.e., social) and internal (i.e., endocrine, psychoactive chemical, etc.) environments are additively or synergistically pathogenic underlie development of the illness in question? If so, how (again) does this approach to differential diagnosis facilitate – or even allow – incorporation of such a possibility?

The conceptual handicaps we impose on our students and residents are not limited to the fallacies inherent in the diagnostic system we teach them; the opportunity cost of precious curricular time and emphasis may be at least as problematic. A major premise of this critique is that the neo-Kraepelinian assumption that psychopathology presents as discrete categorical entities as defined in the DSM is turning out to be inconsistent with the way genes and environments act and interact to produce brain function and dysfunction. And while it may be many years before we have an understanding of these mechanisms that is sufficient to form the basis of a new nosology, we are closer than either the emphasis on the current system, or the near absence in clinical education and training contexts of discussion of etiopathogentic mechanisms, implies.

So does “the state of psychiatric science” dictate major change for the DSM? It does – not because we have the answers, but because we do not. We need a nosology that is sufficiently nimble to be able to incorporate the understandings that will be forthcoming, and sufficiently tentative to allow those understandings to develop among investigators, clinicians, and trainees alike. Taxonomy cannot lead conceptual innovation but can only aspire to reflect it. Perhaps most importantly, it should never retard it. Our current one must be scaled back considerably in both form (i.e., scrapping the multiaxial system) and scope (i.e., acknowledging that not all conceivable permutations of human distress or dysfunction need or should be named). Those diagnostic entities that remain should both describe and help us investigate the phenotypes, etiopathogeneses, prognoses, and treatment responses of the patients so categorized. This set of recommendations is at once radical and conservative, ambitious and austere. The stakes involved demand such audacious caution.

### Commentary: science, conservative by nature

Michael Cerrulo, M.D.

University of Cincinnati Department of Psychiatry

Psychiatry is the last area of medicine to be based almost exclusively on descriptive science. This in itself is not necessarily a criticism as every branch of medicine (and science) must start with a descriptive knowledge of its subject. Psychiatrists need not feel any inferiority to the rest of medicine: rather we have simply chosen the most difficult disorders of the most complicated organ, the brain. Modern psychiatric nosology builds on Kraepelin’s method of diagnosis based on longitudinal history and current symptoms. The DSM-III and IV were refinements in Kraepelin’s diagnostic systems that have proved incredibly useful.

There have been impressive advances in neuroscience in recent decades and an optimist could easily believe that we appear to be on the verge of understanding the etiology of some of the most severe mental illnesses. Yet the reality is that at the current time we do not have this understanding. The future is always notoriously difficult to predict and although we hope answers are just around the corner it may be many more decades before real etiological models can be defined. Therefore it is premature to talk about a paradigm change in the DSM-V and the use of biomarkers and etiologically based definitions of disease when neither exists in psychiatry. Science by nature is always conservative. According to Kuhn [37] each branch of science works within a paradigm that encapsulates the basic understanding of the field. When important new findings occur that can no longer be incorporated into the current paradigm then conservatism is forgotten and a major change occurs in the fundamental principles of the field. The classic Kuhnian example of paradigm charge is the overthrow of Newtonian physics by relativity and quantum mechanics in the early part of the twentieth century.

In Kuhnian terms the current diagnostic paradigm in psychiatry is based on Kraepelin’s method. Unless there is overwhelming evidence to throw out this paradigm then conventional wisdom (supported by the history of science) suggests we make only small conservative changes where necessary. Of course even these small changes need to be supported by good empirical evidence. But even supposing we had more information on biomarkers and disease etiology, would these really necessitate a paradigm change? It seems a stretch to believe lab tests will become the new goal standards that define all mental illness. A more realistic appraisal seems to be that lab tests will be incorporated into the current diagnostic system which will still require information on the current and past course of symptoms.

There are also other more pragmatic worries about any superfluous changes in our diagnostic criteria. Even minor changes in diagnostic criteria could lead to significant changes in the prevalence rates of the respective disorder. Thus public health consequences also need to be considered before each potential change. Then there are concerns about how insurance companies will take advantage of any changes to deny coverage. Finally, changes in many psychiatric diagnoses could have significant legal and social implications (See my essay on the first question for a justification as to why these pragmatic concerns and value judgments are legitimate parts of our disease definition). In total these many concerns suggest we take an extremely conservative approach to changing our diagnostic system.

### Commentary

Andrew Hinderliter, M.A.

University of Illinois Depart of Linguistics

In recent blog posts, Dr. Frances has acknowledged that a serious problem with the DSM-IV’s conservative approach to diagnostic change is “grandfather[ing] in weak links.” In these posts are included responses from myself [38] and Dr. Charles Moser [39] suggesting possible solutions, and Dr. Frances’ responses.

To further thinking on this issue, I will focus on the paraphilias—as did both my and Dr. Moser’s discussions—because this is what my published work regarding the DSM has dealt with and because it illustrates both the severe problems that DSM-IV’s conservative approach to diagnostic change can have in some areas and how the DSM-5 spirit of innovation is not a viable solution.

Psychiatry has a long history of pathologizing sexual variations, a clear case of medicalizing morality. In the US the only variant sexuality to be removed from the DSM has been homosexuality, and this would not have happened without outside pressure from gay liberation forcing the APA to deal with the issue. Several remaining “perversions” illustrate one of the most embarrassing consequences of DSM-IV’s conservative approach to diagnostic change: though a medicalization of morality, it is not even *our morality*. Viewed from the increasingly dominant “between consenting adults” standard, the DSM’s lumping together of cross-dressing and sex with children is downright bizarre, but it should be remembered that in the 1950s, cross-dressing was illegal in many parts of the US. In recent years, some Scandinavian countries have removed sadomasochism, transvestism, and fetishism from their versions of the ICD, but they left in the illegal disorders of sexual preference, making it clear that this is still a medicalization of morality. But at least it is their morality. The DSM’s continued pathologizing of increasingly accepted sexualities will increasingly be an embarrassment to the APA.

If the divergence of the old and the new morality reveals the most ridiculous consequence of grandfathering in the paraphilias, the most pernicious effects are seen where the old and new morality coincide. In the US, 20 states and the federal government lock up certain offenders *after* completing their sentences under Sexually Violent Predator (SVP) laws (mostly passed after the publication of DSM-IV) which require a diagnosis of some mental disorder, the paraphilias and antisocial personality disorder being the most commonly used. While the APA has historically been strongly opposed to SVP commitment, keeping the paraphilias in the DSM supports and maintains this.

Despite the APA’s historical opposition to SVP commitment, the DSM-5 Paraphilias Subworkgroup is demonstrating all-out support, revealing the folly of DSM-5’s approach to innovation. In their literature reviews, lack of concern for clinical utility is almost tangible. They acknowledge that these diagnoses hurt “patients” and discourage them from being honest, but they show no interest in changing this. (I strongly encourage readers to look at “APA Guidelines Ignored in Development of Diagnostic Criteria for Pedohebephilia” by a member of a patient advocacy group.) The Paraphilias Subworkgroup is proposing to add paraphilic coercive disorder (PCD) to the DSM, which would legitimate the current rogue use of paraphilia NOS: nonconsent in SVP commitment. The defense for adding PCD to the DSM has been given in two reports by advisors of the Paraphilias Subworkgroup: one written by the Treatment Director at an SVP commitment center, and the other by an SVP prosecutor. Their newly proposed diagnoses are actually being field tested in SVP commitment centers, leaving no doubt about the purpose of these diagnoses: they are not about helping patients, but bringing about their demise.

Following a debate about these at the American Academy of Psychiatry and Law about the proposed addition of hypersexual disorder, PCD, and expanding pedophilia into pedohebephilia, a non-binding vote was held: 31–2 against the second and third and 29–2 against the first. At the International Association for the Treatment of Sexual Offenders, a non-binding vote was held regarding the expansion of pedophilia. The outcome: approximately 100–1 against.

A huge problem with DSM-5’s approach to innovation is that, evidently, it only requires consensus among an extremely small group of people. Taking a conservative approach to diagnostic change can prevent this (i.e. do damage control) but at the price of grandfathering in even diagnoses that are clearing causing far more harm than good. If psychiatry is a legitimate branch of medicine aiming to alleviate human suffering rather than a means—masquerading as medicine—of detaining undesirables, the APA must get its house in order. Neither the DSM-IV nor the DSM-5 approach to diagnostic change can do this.

### Allen Frances responds: To be or not to be conservative

Dr Phillips usefully frames the conservative issue along two orthogonal dimensions – 1) what is most likely to further the validation of psychiatric diagnoses and scientific advances in the field? and; 2) how to deal with value judgments that may not lend themselves to scientific validation.

Neither question has a simple right answer, but I think conservatism trumps when one takes all the risks and benefits into account.

The easiest question is whether DSM should be changed to further scientific advancement. The obvious answer would be "of course, but": 1) We don't really know which changes would improve science; 2) Changes may retard science by making previous findings incompatible with new ones; 3) DSM has a huge clinical responsibility that must take priority – it cannot include diagnoses to increase NIMH funding if such inclusion will also simultaneously result in excessive, false positive diagnoses and over use of unnecessary and potentially harmful medication.

I come away convinced that the DSM-IV conservative position was absolutely right in trying to hold the line against the rampant push for diagnostic inflation. Knowing what we know now from subsequent events, there is reason to question all three of the DSM-IV decisions that strayed from strict conservatism – the inclusion of Bipolar II and Asperger's, and a rewrite of the criteria for ADD. Each decision was made for excellent reasons that withstand the test of time – but each led to an unexpected frenzy of diagnostic enthusiasm that far overshot the mark beyond the useful purpose of the DSM-IV intention.

I can't say it was a mistake to include these innovations in DSM-IV, but I also can't say that their inclusion has clearly resulted in more good than harm. We failed to anticipate the outside forces that can greatly amplify the impact of any DSM decision, especially the power of drug company marketing to create the fads of Bipolar and ADD diagnosis. We never envisioned that diagnoses of autistic disorder could explode twenty-fold.

Any change in DSM can have powerful unintended consequences that cannot be predicted or prevented. The written word of DSM is likely to be loosely interpreted and misapplied in general practice. Because those who write the words can exert little control over later practice, the criteria should be as explicitly restrictive and precise as possible.

I am much less confident in defending DSM-IV conservatism when it prevented the sunsetting of questionable and potentially quite harmful diagnoses. Hinderliter [38], Phillips [40], and Moser [39] make a persuasive case that the high evidentiary requirement for change that governed DSM-IV decisions should have been relaxed for those problematic diagnoses that had been grandfathered into DSM under looser previous standards.

Our rationale was reasonable, even if its results were not always palatable. To keep the diagnostic system from expanding wildly, we established extremely high thresholds for change in DSM-IV. Substantial scientific evidence was required for changes in either direction – those that would add to the reach of the system, but also those that would subtract from it. We feared that without clear and high scientific thresholds, changes would be arbitrary, destabilizing, and subject to personal whim.

This requirement did indeed permit the grandfathering of diagnoses that would not have met the new much higher standards for new suggestions.

I agree completely with Mr Hinderliter [38] that this conservative approach has had unfortunate consequences particularly in the paraphilia section. It also prevented us from taking a stronger stand against the general problem of diagnostic inflation.

We did not alter grandfathered suggestions because we did not know how to do this in a way that would not be arbitrary, controversial, and subject to whim. Dr Phillips [40] suggests a solution that works in the abstract, but I don't know how you would implement it in practice. I see no obvious way of making the discrimination that is central to his suggestion – between ‘real’ psychiatric disorders that lend themselves to scientific judgments versus the nondisorders that call for value judgments. One man's ‘real disorder’ is another man's value oriented "non disorder" and how is one to tell them apart?

Dr Moser [39] has an extremely appealing alternative solution. Keep the grandfathered diagnoses in the DSM, but (using the techniques of evidence based medicine) give ratings that would guide consumers about the confidence one can place in each disorder. This might be a wonderful advance, but as always the devil is in the details.

**Response to Dr Waterman**: I agree with much of Dr Waterman's critique of DSM-IV, but think he underestimates its value and more to the point offers no alternatives or consideration of the risks and benefits of making changes.

**Response to Dr Cerrulo:** I agree completely.

**Response to Andrew Hinderliter**: I agree completely with Mr Hinderleiter on the recklessness of DSM-5 in suggesting new and unproven paraphilias that would encourage the already grave existing misuse of psychiatric diagnosis in the legal system. He and I might however part company on the wisdom of removing existing diagnoses from the nomenclature – particularly Pedophilia and Antisocial Personality Disorder. He might see these as clearcut value questions with an obvious right answer. I find the situation more complicated and difficult. Both diagnoses have a clinical tradition that stretches back more than a century, a substantial body of research, and some clinical and prognostic utility. I am not convinced they cause more harm than good, and tie scores (or near tie scores) should in my view always go to incumbency. But it would be valuable to have the discussion re sunsetting these and other potentially weak diagnoses accompanied by a thorough evaluation of the evidence.

### Question #4: is pragmatism practical?

What roles do science and pragmatism play in the construction of DSM-5? Does our science allow us to make major decisions on a scientific basis? What role do pragmatic considerations play, both when the science is strong and when the science is weak?

## Introduction

The introduction to this question must begin with the same point that began the introduction to the previous question: the agreement on the part of most discussants regarding the weak scientific status of the DSM-IV diagnoses. In the case of the previous question, the discussion set off from that general agreement to the further question as to whether the current state of psychiatric science leads one to proceed cautiously or vigorously in developing DSM-5. With Question 4, we question whether, in the current situation of weak science – or even in an imagined situation of strong science in the future – pragmatic decisions should play a role in the construction of the manual.

Let us begin with an understanding of how we will use the word ‘pragmatic’. Omitting a long discussion of pragmatism in philosophy as developed by Peirce, James, and their heirs and adherents, we will understand ‘pragmatic’ in our discussion to refer to the *practical consequences* of a diagnostic construction. We thus make a distinction between the truth status of a diagnostic construct – its scientific validity – and its consequences or effects (again, acknowledging that in the pragmatic tradition, the effects will be linked to the truth status). The potential consequences are many and varied: whether the diagnostic construct, with its set of diagnostic criteria, will expand or limit the population covered by the construct, whether (relatedly) it will create more false positives or false negatives, whether it will create more or less zones of rarity between one diagnosis and another, and whether (again relatedly) it will separate off the diagnosis from normality or tend to merge it at one end into normal feeling and behavior.

We need to add that all of the above effects are subordinate to a single overarching effect - the effect of change on patient welfare. For this effect our shibboleth is the Hippocratic maxim: *Primum non nocere* - First, do no harm.

The question arises: is there a conflict between science and pragmatism in the DSM? In my opinion there is not. The DSM does and must involve both. The DSM must use the science that is available, but it must also make countless judgment calls that are not grounded in solid empirical evidence—and surely it makes sense to consider practical consequences in doing the latter. First, there is the question as to whether a condition should be listed at all in the DSM as a disorder. As discussed in the above question, various groups of “paraphilics,” for instance, have protested as did homosexuals decades ago, the pathologic status of their sexual difference. To invoke a provocative example, what is the empirical evidence that pedophilia is a psychiatric disorder, rather than simply a socially repellant behavior? A similar question, raised and studied by John Sadler, involves the bad-conduct disorders such as Conduct Disorder and Antisocial Personality Disorder [41,42].

Second, once a disorder is admitted into the DSM, we make countless non-empirically based decisions about the structure of the diagnostic criteria—what they are, how many there are, how many are needed to declare the diagnosis, etc. All such decisions utilize the available empirical evidence, but they are hardly dictated by that evidence. And where judgment comes in, so does consideration of consequences. In the case of virtually every diagnosis with diagnostic criteria, increasing the number of required criteria limits the population and creates false negatives, while decreasing the number of required criteria has the opposite effect of expanding the population and creating false positives.

Let us focus on an example: the criteria set for a major depressive episode. The requirement of only 2 weeks of symptoms, and only 5 of 9 of the criteria, may cast too wide a net, create a huge population of patients with this diagnosis, and bring in too many false positives. Do we have a scientific basis for the judgment that someone with one week of the required criteria does not suffer from a major depressive episode but that someone with two weeks of the criteria does suffer from a major depressive episode? If not, then we have to decide whether we are more concerned with overdiagnosing or underdiagnosing major depression.

Then there are the much disputed sub-threshold conditions, with most of the attention going to

Psychosis Risk Syndrome, now renamed Attenuated Psychosis Syndrome and Disruptive Mood Dysregulation Disorder (previously called Temper Dysregulation Disorder with Dysphoria). The first is considered a prodromal schizophrenic condition and the second a prodromal bipolar condition. In each case we debate the pragmatic consequences of sticking these diagnostic labels on young individuals (in the first case, even with evidence that only a third of the so-labeled group will emerge as schizophrenic). What are the practical consequence of introducing or not introducing these diagnostic categories?

Finally, there is the debate over whether we will be adversely expanding the population of bipolar patients, and expanding the use of potentially harmfully medications, by shortening the number of days of hypomania required to diagnose bipolar disorder. Science will certainly play a role in this debate, follow-up studies showing whether patients with brief hypomanic episodes become more clearly bipolar, and whether early intervention decreases progressive illness.

In none of these examples does there appear to be a conflict between science and pragmatism, and in all of them both science and pragmatism play a role in the development of the diagnostic construct.

### Commentary: the DSM and “do no harm:” is a radical pragmatism sufficient?

Warren Kinghorn, MD

Duke University School of Medicine

In discussing the first question, Dr. Frances, in a helpfully candid glimpse into the politics of psychiatric diagnostic classification, classifies himself as the second of the five umpires in the “epistemologic game.” This categorization would suggest that he is a realist with regard to the ontology of “mental disorder” – for the second umpire, the balls and strikes clearly exist independently of the umpire’s judgment– and in fact there are glimpses of realism in Frances’ account, as when he holds that the NIMH Research Domain Criteria (RDoC) project rather than *DSM* might “lead the future charge in understanding psychopathology.” But this ostensive commitment to diagnostic realism is somewhat undercut by Dr. Frances’ pragmatic and constructivist assertion that “mental disorders don’t really live ‘out there’ waiting to be explained. They are constructs we have made up . . .” Furthermore, for Frances it is pragmatic, not realist, commitments which should guide revisions to diagnostic criteria: *DSM* should be revised not when new mental disorders “out there” are recognized (for how, after all, would we know a “mental disorder” if we saw one?) but rather when the consequences of a revision are likely to provide benefit to patients and, above all, will do no (anticipated) harm – the position of umpire 4. *DSM,* for Frances, serves and should serve as a regulatory and even disciplinary document demarcating limits for the appropriate extension of psychiatric technology and for the appropriate use of psychiatry by particular interests (such as the state).

Frances is surely correct regarding the social function and power of *DSM* and regarding the need to approach potential revisions with extreme care. But his insightful account begs the important question: *who should decide?* Who should decide what “mistakes” and “problems” are, or what “mental disorder” is, or what constitutes “harm,” or what would render *DSM* “useful?” Should patients decide? Should individual psychiatrists decide? Should the Task Force decide? And on what grounds? And how would we know if the judgments of any of these potential “deciders” were shaped, subtly and unconsciously, by particular forces such as pharmaceutical marketing, consumer-driven ideals of beauty and success, gender stereotyping, and so on? Can psychiatric diagnosis ever extricate itself definitively from Foucauldian and Szaszian critique? It is difficult to see how Frances’ pragmatism can ensure that diagnostic revisions will “do no harm” if “harm” is itself a contested category.

If, as Frances argues, efforts to establish a consensual and non-tautological account of “mental disorder” are likely to fail, there would seem to be no way around these questions. Psychiatric diagnostic classification, that is, must be understood as a pragmatic and tradition-constituted enterprise in which individuals and groups with particular interests interpret research data (itself compiled and reported by individuals with particular interests) in such a way as to shape the use of psychiatry and psychiatric technology in accord with these interests. This recognition should, at the very least, provoke humility and non-defensive soul-searching among those tasked with revising the *DSM,* since biases and moral failures in the “deciders” would very likely become manifest in their nosological decisions, and the ongoing cultural acceptance and use of the *DSM* hinges on the ongoing public credibility of these “deciders.” It is no wonder that the *DSM-5* architects, in the face of much work in the contemporary philosophy of psychiatry, continue to speak in realist terms about syndromes “actually present in nature” and a nosology which “[carves] nature at its joints” [32, p. 645–8]. In the absence of a narrative of progressive scientific discovery, would the social consensus regarding the usefulness of the *DSM* continue to hold?

### Commentary: the ethical significance of pragmatic science for DSM

Douglas Porter, M.D.

New Orleans, LA

The relationship between pragmatics and the science of nosology is complex. But, for reasons that are ethically significant, it is important to clarify that nosology is always already practical science. Nosology cannot be understood in complete distinction from pragmatics. Practical therapeutic concerns permeate the science of nosology through and through, and there is no way to make sense of nosology without keeping these concerns in view. The practical concerns of nosology do not stand in contradistinction to a scientific concern for objectivity. On the contrary, the practical therapeutic concerns that ground the science of nosology ground the demand for scientific rigor; the type of scientific rigor that presents a safeguard against idle empirical assertions that are more akin to acts of wish fulfillment or confabulation than a faithful reckoning with the world in which we live. While the practical nature of nosology demonstrates the necessity of objectivity, it also demonstrates that the concern for objectivity, in and of itself, is insufficient. A collection of undeniably objective yet arbitrary facts would hardly make for a valid classificatory schema. There must be some additional normative measure of the salience of the objective data. The notion of practical science therefore encompasses the concern for objectivity but extends beyond it to issues of relevance that are best justified in terms of an ethical framework. Justifying the validity of diagnostic constructs is not merely a matter of objectivity. This fact causes no end of semantic difficulty because “validity” has become virtually synonymous with “objectively true” in the discourses of the sciences.

At times in the history of medicine the careful description of syndromes has been associated with the discovery of singularly determining etiologies. There is no question about the pragmatic therapeutic significance of these discoveries. The influential criteria for validating psychiatric diagnostic constructs outlined by Robins and Guze [34] are compatible with the assumption that the signs and symptoms of mental disorders cluster together in syndromal fashion due to an underlying mechanism that uniquely determines the course of illness regardless of context. This notion of “validity” is thoroughly entrenched. Kendell and Jablensky [33] note that when diagnostic categories convey information about outcome, treatment response, and etiology they may be recognized for their “utility”. But, these categories with utility must still be regarded as invalid if they fail to reflect the type of context independent, singularly determined entities that would clearly distinguish a particular disorder from normality and all other disorders. Indeed, while much ado has been made about the significance of a shift from categorical to dimensional diagnoses, it is apparently possible to make this shift while retaining the firm conviction that valid disorders will carve nature at the joints [32]. The dichotomy between “valid” disorders and disorders with “utility” is misleading. It would appear to indicate that this particular notion of validity transcends pragmatic issues of utility. But, as I indicated earlier the justification of validity in nosology necessarily extends beyond empirical issues to normative issues of relevance. This burden of justification extends to the notion that “truly” valid disorders have a singular essence that uniquely explains their form of pathology. If singularly determining etiologies existed for mental pathology they would hold powerful explanatory value and great utility. But, it is unclear to me that it is “valid” to conceive of mental disorders primarily in terms of simplistic singularly determining etiologies when accumulating empirical evidence indicates that the signs and symptoms of mental disorders develop in a highly contingent manner through complex interactions between biological factors and the environment. As Kendler [43] notes, social and cultural factors and the agency of the person with illness become entwined in their own right as pertinent, but not singular, explanatory factors.

Ultimately issues of validity will be decided not by empirical data alone, but by measures of the relevance of the empirical data. It is for this reason that I spoke of the ethical significance of remembering the practical nature of our science. Preoccupation with objective data that hold no relevance for the science of therapeutics makes for nosology that may be objectively valid but fails to be ethically valid because it fails to maintain its practical relevance. The signs and symptoms associated with DSM disorders may have been recognized as valid only insofar as they reflected a singular underlying etiology, but they carried ethical significance for different reasons. The clusters of signs and symptoms detailed in the DSM kept disorders relevant by keeping them firmly tied to the real world concerns of people dealing with mental illness. Insisting that it is only valid to proceed with diagnoses that submit to powerful singular forms of explanation when real world concerns do not submit to such explanation seems akin to looking for lost keys on the street where the light is good, instead of looking on the street where the keys were actually lost. To insist upon a unitary explanation when it does no justice to the complexity of the phenomena of concern can undermine the therapeutic relevance of nosology by prematurely marginalizing pertinent levels of explanations and the types of therapeutic intervention for which they call. To insist that issues of validity transcend practical matters of utility belies the fundamental issue of relevance for the practical science of nosology and the ethical form of normative argument ultimately required to justify its validity.

### Commentary

Joel Paris, M.D.

McGill University Department of Psychiatry

While in principle, science should be the basis of any diagnostic system, DSM has been seduced by the illusion that advances in neuroscience provide empirical validity for a new system. In reality, we do not know whether conditions like schizophrenia, bipolar disorder, or obsessive compulsive disorder are true diseases.

Current diagnostic concepts tend to be more pragmatic than scientific but have become reified with constant use. DSM-5 needs to re-emphasize that its classification can only be provisional.

In spite of all the progress that has been made in neuroscience over the last few decades, we are no closer to understanding the etiology and pathogenesis of mental disorders than we were fifty years ago. Applying scientific findings to the understanding of psychopathology is a complex task that could require many decades more. The idea that is often promulgated, that breakthroughs are just around the corner, are at best hype and at worst seriously misleading. Thus, DSM has no choice for the foreseeable future but to continue with a classification system based on phenomenological observation. For that reason, more effort should be expended on psychometrics and discriminant vaidity of measures, and less on chimerical searches for neurophysiological or neurochemical specifics. While the establishment of biological markers must remain a long-term goal, DSM should be written for 2013, not for 2063 or 2113.

### Commentary

Joseph Pierre, M.D.

UCLA Department of Psychiatry

An ideal DSM would be based upon known “biopsychosocial” etiologies and have wide-ranging contextual utility, but until that ideal is achieved, competing utilities must be carefully balanced. If, for example, scientific discovery since 1994 had yielded the genetic basis for several major psychiatric disorders, a new DSM-5 would clearly be justified. “Pragmatic” considerations about the implications to clinical therapeutics or public health policy would then have to follow. Instead, with the promise of etiologically-based diagnoses still unrealized despite scientific progress since 1994, all we really have are pragmatic considerations about DSM revisions (or in arguing against revisions altogether). Note that this isn’t quite the same as saying that pragmatism is more important when science is weak and vice-versa, but rather that pragmatism should always be optimized given the existing state of scientific knowledge.

The case of “prodromal psychosis” or “psychosis risk syndrome” offers a good example of the challenges in balancing competing pragmatic considerations and contextual utilities in the face of a theory-driven (as opposed to a fact-driven) proposed DSM revision. Current research efforts to develop effective primary and secondary preventative strategies for psychosis are ongoing and worthwhile. However, research criteria for subjects included in those studies have already been developed and standardized, such that further scientific progress doesn’t require a DSM revision. If it were the case that these research criteria were fully validated, with a substantial majority of identified at-risk individuals inevitably “converting” to psychosis, then creating a new category of “psychosis risk syndrome” would be justified, despite certain pragmatic difficulties (e.g. not knowing how to best treat the condition). In that case, pragmatic considerations would have to follow from the newfound ability to reliably predict the development of a psychotic disorder. In reality though, “false-positive” rates in studies of “prodromal psychosis” have been as high as 84% at 2-year follow-up [44], such that together with the lack of knowledge about the risk/benefit profiles of putative interventions, there is a solid case against inclusion [44,45] in a DSM whose “highest priority has been to provide a helpful guide to clinical practice [46].” Proponents of the inclusion of “psychosis risk syndrome” in DSM-5 view the data more optimistically [47], and argue that DSM-5 inclusion would be helpful to promote further funding of research in this area [48]. These pro and con arguments are all based on the same scientific evidence, but touch on different aspects of pragmatism and contextual utility (i.e. will inclusion in DSM-5 be most useful for patients, clinicians, or researchers?). Since contextual utilities are often conflicting, any final decision about inclusion will inevitably be based upon pragmatic choices that prioritize different risks and benefits.

The pragmatic concern that most intrigues me for DSM-5 is the larger issue of “diagnostic expansion” in psychiatry [49]. The number of psychiatric disorders has been increasing since the first DSM, and many of the most controversial newly proposed disorders for DSM-5 (including psychosis risk syndrome) are those that seem to be encroaching on normal behavior. This kind of expansion is occurring for three main reasons. First, we increasingly recognize that psychiatric symptoms exist on a continuum with normal experiences (e.g. anxiety, sadness, aggression, age-related cognitive decline, and even psychosis). Therefore, the boundary between pathology and normality is inherently difficult to define. Second, to an individual or clinician, whether or not an individual meets criteria for a DSM disorder is less important than whether they are suffering. Indeed, the presence of suffering is usually regarded as a defining feature of “clinical significance” and therefore pathology in mental illness. As a result, if an individual is in distress and help-seeking, then treatment of some kind will usually be offered, and having a diagnosis to justify that intervention is incentivized. Third, treatment strategies, whether psychological or pharmaceutical, have the potential to help people with clearly pathological as well as “subclinical” or “subthreshold” symptoms alike. All of these realities drive a psychiatric market that is increasingly shifting towards neuroenhancement, with increasingly blurry distinctions between pathology and normality.

This shift has already prompted concerns about “disease mongering” [50] and other thorny ethical debates [51,52], including whether the pursuit of happiness, which is always relative, is the proper goal of psychiatry. For good or bad, I view the shift towards further diagnostic expansion and neuroenhancement as inevitable so long as the technology to make people “better” exists. However, to what extent this trend should be sanctioned in the DSM requires careful thinking by developers about pragmatic implications in different contextual arenas. DSM’s main utility may be as a clinical guide, but the reality is that DSM diagnoses are widely used (and misused) for non-clinical decisions.

### Allen Frances responds: practical pragmatism

I often get asked this question – whether practical consequences should play an important role in DSM5 decisions. My quick answer is a very emphatic yes – pragmatic concerns must play a central role in shaping any DSM.

Why is this the case? DSM is an official system of classification that has a huge (perhaps excessive) influence on how everything works in the mental health world – who gets diagnosed, how they are treated, who pays for it, whether disability is appropriate, and whether someone can be involuntarily committed, released from legal responsibility, or sue for damages. DSM also has a diverse influence on public policy – directly or indirectly influencing things as varied as the way scarce treatment and school resources are allocated, the impact of medication on the obesity/diabetes epidemic, and how sexual offenders are (mis)handled in the legal system.

Ever since the introduction of DSM-III, the DSM system has been a great promoter of psychiatric research and the principal means of translating across the clinical/research interface. But, DSM is decidedly first and foremost a clinical document, with its other uses being important, but definitely secondary. As an official diagnostic system, DSM is not meant to place its highest priority on promoting or facilitating the latest in research ideas. Because it has such a powerful influence on real life (and occasionally even life or death) decisions, DSM can't ignore its practical consequences – intended or unintended. It has to be workaday – trying very hard not to make mistakes that will hurt people, rather than having fancy but untested "paradigm shifting" ideas that almost always wind up doing more harm than good.

Which brings us finally to the question of how best to make DSM decisions. Much has been written about the ‘validators’ of psychiatric diagnosis and how they should influence DSM. The problem is that available information on the validators for most diagnoses is usually equivocal and inconsistent – validators never reach out, grab you by the throat and say "Do it this one way or the science gods will be displeased."

To my mind, by far the most important validator is how will any decision help or harm patient care, given the foreseeable circumstances under which it will be used. Let's go back to how this practical, common sense approach works for the boundary between unipolar and bipolar disorder. Start with the facts that there is no biological test to make the distinction and no certain way to know what the appropriate ratio should be among mood disorder patients. We do know one important fact. The ratio of bipolar diagnoses at least doubled since the introduction of Bipolar II in DSM-IV and the extraordinary drug marketing campaign promoting antipsychotics and mood stabilizers. This has undoubtedly helped some people and harmed some others – the exact extent of each is unknown and perhaps unknowable. But my bet is that this is a fad that has overshot – they always do. I would assume that anyone now presenting with anything suggesting equivocal bipolar disorder is much more likely to be overdiagnosed and overtreated than to be missed. Close watchful waiting in doubtful cases beats rushing in with potentially dangerous meds.

DSM-5 should always take full account of the risks, not just the benefits, of getting a diagnosis and factor in the side effects and complications of the real world treatments (usually medication) that will follow. Those working on DSM-5 must take responsibility for the practical consequences that their decisions will have on peoples' lives.

Many people are troubled by the fact that an evaluation of practical consequences necessarily plays such an important role in making DSM decisions. They would prefer that these be settled somehow more ‘scientifically’. What they fail to appreciate is that the scientific data underlying descriptive psychiatry never provides a clear and unique right answer about where to set diagnostic boundaries. All else being more or less equal scientifically (which it almost always is for the kinds of boundary questions we are discussing), by far the most important deciding factor should always be: is this change (as it will be applied in the average expectable practice environments) more likely to help or hurt patients? Or in other words – what is more dangerous here, missing the diagnosis or overdiagnosing.

Of course, these decisions are always subject to differing interpretations of the available data and the possible extrapolations from it – but this is what a thorough risk/benefit analysis is all about. We have to live with the fact that here, as in so much of medical decision making, the science can only take us so far and never jumps off the statistical tables to provide us with the single, right answer.

**Response to Dr Kinghorn:** Dr Kinghorn’s extremely penetrating critique of my position – that my pragmatism lacks normative values and a vouchsafed method – cuts straight to the heart of the matter and is devastatingly accurate and impossible to dispute. His critique is so telling I will quote its central portions again to provide the emphasis it deserves: "But his insightful account begs the important question: who should decide? Who should decide what ‘mistakes’ and ‘problems’ are, or what ‘mental disorder’ is, or what constitutes ‘harm’, or what would render DSM ‘useful’? Should patients decide? Should individual psychiatrists decide? Should the Task Force decide? And on what grounds? And how would we know if the judgments of any of these potential ‘deciders’ were shaped, subtly and unconsciously, by particular forces such as pharmaceutical marketing, consumer-driven ideals of beauty and success, gender stereotyping, and so on?"

The essential problem of utilitarian pragmatism is that it often lives case by case, without clear external value guidelines of the good or even the best methodologies for establishing what those guidelines should be. Suppose a drug for schizophrenia improves life, but in the process shortens it – who decides how the utilities should play out? In deciding whether to add a new diagnosis for "psychosis risk syndrome," one pragmatist may worry more about the lost benefit for false negatives of not having the diagnosis; another (I think wiser) pragmatist about the treatment burden on false positives if it is included. The Benthamite utilitarians tried to solve this conundrum with "the greatest good for the greatest number" and developing metrics for "good" is now part of behavioral economics. But as Kinghorn puts it, the basic question is often begged – who decides the values, goals, and methods of utilitarian pragmatism and how?

Back to Dr Kinghorn's telling words: "It is difficult to see how Frances’ pragmatism can ensure that diagnostic revisions will 'do no harm' if 'harm' is itself a contested category. If, as Frances argues, efforts to establish a consensual and non-tautological account of 'mental disorder' are likely to fail, there would seem to be no way around these questions. Psychiatric diagnostic classification, that is, must be understood as a pragmatic and tradition-constituted enterprise in which individuals and groups with particular interests interpret research data (itself compiled and reported by individuals with particular interests) in such a way as to shape the use of psychiatry and psychiatric technology in accord with these interests. This recognition should, at the very least, provoke humility and non-defensive soul-searching among those tasked with revising the DSM, since biases and moral failures in the 'deciders' would very likely become manifest in their nosological decisions, and the ongoing cultural acceptance and use of the DSM hinges on the ongoing public credibility of these 'deciders’."

Right on. But to where? The DSM's have come to assume enormous (probably too much) influence in widely diverse decisions that impact greatly on public health, the distribution of scarce mental health and school resources, and even the protection of constitutional rights. The scope and strength of influence of DSM has grown far beyond what anyone could have envisioned thirty years ago. The American Psychiatric Association has sponsored the DSMs for sixty years, taking on the task originally because no one else wanted to be bothered with anything so insignificant. It seems clear now that the importance and scope of the psychiatric diagnosis has outgrown its being comfortably nested within a single professional organization. The sorting of different values and weightings in making tough pragmatic choices require much wider consensus.

 If not the American Psychiatric Association, then who should be responsible for future revisions in the diagnostic system? There is no clear right answer. My best (but far from perfect) choice would be the National Institute of Mental Health. NIMH would bring a far broader view to the task and be less burdened by publishing concerns. But NIMH also has limitations. It would tend to be too research-focused, less sensitive to practice concerns, and not necessarily representative of larger public policy and forensic issues. So my choice would be NIMH supervision of a very inclusive and transparent process.

None of this really answers Dr Kinghorn's fundamental point. Nor can it be answered. Given the current state of psychiatric knowledge, there are rarely clearly right choices based on a cut and dried science base, and the proper course of pragmatism is often in the eye of the beholder. The safe play is to be aware of risks and potential blind spots and to build in a lot of checks and balances. In an uncertain world, your worst critics are often ultimately your best friends.

**Response to Dr Porter**: Dr Porter makes the important point that diagnostic decisions must necessarily be based not only on science and pragmatics, but also sometimes on difficult ethical tradeoffs. This is especially the case when, as is often true, the science is inconclusive and the practical consequences are both positive and negative. The best examples are questionable new diagnoses (see Dr Pierre on "psychosis risk") that may be very helpful for some people, but also may trigger the massive overuse of potentially dangerous medications for those who don't need them.

Real world and ethical concerns must always be given a great deal of weight especially when excessive reliance on a weak science base may result in disastrous, unintended practical consequences. The most egregious current example of ignoring this common sense precept is the dangerous over treatment of a wildly expanded misdiagnosis of childhood bipolar disorder.

**Response to Dr Paris:** Dr Paris is rightly concerned that some voices in psychiatry have been seduced by the neuroscience revolution. They attempt to borrow its authority to make excessive claims for the scientific underpinnings of clinical psychiatry generally, and for DSM-5 specifically. Such true belief in "science" (and over-selling its contribution) helped germinate the misplaced ambition of DSM-5 to effect a "paradigm shift" in psychiatry. Unfortunately, the rapid expansion in our understanding of brain function has had little impact on either psychiatric diagnosis or clinical practice. Indeed there is some useful science available to guide diagnostic decisions, but it is often sorely incomplete in answering the crucial questions that must be included in any serious risk benefit analysis: what effect will this change have on the overall rates of the disorder and on the rates of false positive diagnosis? How effective will treatment be? How harmful will treatment be? What is the natural course without treatment? How do we balance the possible benefit of diagnosis and treatment for true positives versus the potential harmful effects for everyone, but particularly the false positives. Lacking precise answers to these questions, decisions must be based on best guesses on practical implications and "do no harm" caution – not an appeal to an often incomplete and uninformative "science."

**Response to Dr Pierre**: While agreeing completely and enthusiastically with Dr Pierre's penetrating analyses, I do have one disagreement with his conclusions. Dr Pierre sees performance enhancing, cosmetic psychiatry as basically inevitable and potentially useful – already evidenced in current liberal prescribing habits and certain to expand much further as new psychiatric diagnoses (and a lowering of thresholds for existing ones) expand the realm of mental disorders and shrink whatever is left of normality. End result – performance-enhancing pills will likely become increasing ubiquitous.

I agree with Dr Pierre's prediction of the bright future of cosmetic psychiatry and the profits it will afford to the drug industry. But I feel a strong need to oppose this trend. My first objection is on practical grounds of efficacy and safety. Most of the perceived performance benefit to be gained from psychotropic medication by those who are mildly ill or not ill at all will probably be due to illusory placebo effect – but the side effects, cost, stigma, and loss of a personal attribution for success will be quite real. Often harmful treatments based on all sorts of nostrums and snake oils have always been a part of medicine and will find their grand revival in cosmetic psychiatry.

On more technical and professional grounds, our current FDA regulatory approach to drug approval requires there be an indication for treating a definite disorder, not for enhancing a skill or for recreation or for a pick me up. If, as a society, we choose instead a "Brave New World" approach of ubiquitous medication management, then this should come only after wide discussion of the profound policy implications. Cosmetic psychiatry should not result from the combination of diagnostic creep (suggested by well-meaning but misguided experts in psychiatric diagnosis) and aggressive marketing promoted by much less well-meaning drug companies. If, after adequate consideration, our society decides that everyday performance enhancement is the way to go, I would personally disagree but accept the policy change. My point is that a decision of this consequence should not rest with any group of medical professionals or with the drug companies.

## Conclusion

Allen Frances’s response to the third question – whether to take a conservative or activist attitude toward change in the DSM – is consistent with his responses that preceded it. Put simply, if current science does not match well with the existing DSM categories, and altering the categories will not improve that match, why change them? Why not leave them alone until science, as possibly with the NIMH Research Domain Criteria (RDoC) project, offers us guidance as to how to change them? And this argument regarding the existing categories can be made in bold for the proposed new categories. With minimal scientific foundation they continue to escalate the medicalizing of human difference; and in the case of sub-threshold conditions like Attenuated Psychosis Syndrome, they increase the ranks of false-positive diagnoses and the exposure of non-ill individuals to powerful psychotropic medications.

Frances is less clear about his position when it involves diagnoses such as the paraphilias that are more value-laden and dependent on the judgments of public morality, and whose status as psychiatric disorders may not be decided by future science. In defense and explanation of DSM-IV, he offers the historical context in which he and his Task Force felt it was important to maintain the high-threshold standard for change in the case of all diagnoses. He acknowledges that this may have resulted in leaving diagnoses in place that would better have been “sunsetted.” He ends with a useful proposal: although in the case of controversial, value-laden diagnoses like antisocial personality disorder and pedophilia we may not be able to decide on their validity in a strictly scientific way, we could try to find a way to measure whether leaving them in the nosology does more good or harm.

Among the commentators, Scott Waterman and Michael Cerullo are striking in that they end in such different conclusions from the same starting point. They both begin with recognition that the DSM categories don’t match contemporary science. Cerullo concludes, like Frances, that this is a warrant for being conservative with changes in the DSM categories until their scientific status is clarified. Waterman draws the opposite conclusion that the categories should be altered to reflect and facilitate ongoing research. Although Waterman’s recommendation to eliminate the axial system and pare down the number of categories is certainly arguable, Frances still questions whether, in the current atmosphere of inadequate science and all the other proposed changes for DSM-5, altering the current DSM will do more harm than good. Frances also remarks on Waterman’s underappreciation of the current manual, but in defending the manual he does not address Waterman’s specific concerns about the difficulties of teaching the DSM in its current form. For my part, given Waterman’s severe critique of his chosen example, DSM-IV major depression, I would like to see his vision of a diagnostic construct of major depression that would accomplish all that he would like from a DSM revision.

In his commentary Andrew Hinderliter takes up the other side of this question, the value-based diagnoses. He makes a cogent argument for questioning and removing some of the paraphilias.

The fourth question, that of pragmatic considerations in developing a nosology, easily connects for Frances to the questions thus far discussed – and adds a further dimension to them. In their current form the diagnoses are shaky creations with uncertain scientific backing; they are a motley group that can’t be neatly grouped together around one clear definition of mental illness; and we have already cautioned that in this state of affairs we should be guarded both in modifications to the existing categories and in introduction of new categories. Let’s now add that in all of this discussion - how we think of diagnoses, how we define them, and how we change them - we should pay great attention to the consequences of our actions on the individuals receiving the diagnoses. The first and last principle here is: First, do no harm. Thus, when considering change to the nosology, we should consider the relevant science, but we should also consider the practical effects on patients. Imagined conflicts between science and pragmatism usually evaporate in the face of scientific uncertainty that is balanced by pragmatic considerations. For an easy, uncontroversial example that I described above, major depression requires two weeks of symptoms and five of nine diagnostic criteria. Lengthen the required period of active symptoms from two to four weeks or expand the needed number of criteria from five to six, and you have reduced the population, inevitably creating fewer false positives and more false negatives. Science will have a voice in such a decision, but will never get it exactly right, because there is no ‘exactly right’. A risk/benefit analysis involves practical considerations of false positives and negatives, along with our differing tolerance for each.

The four commentators are all in broad agreement with Frances that practical, pragmatic considerations play a role in the construction of psychiatric diagnoses, both in the current state of our inadequate science, and at a future point when the science is more secure. Warren Kinghorn leads off with a provocative question that captures our respondent’s attention: namely, in this exercise in assigning priorities to what practical considerations will be highlighted in a particular diagnostic construct, who will decide? In questions that can’t be answered with simple empirical evidence, will we turn the decision-making over to the Task Force, the APA Assembly, a specially appointed committee, the patients, whom? It goes without saying that there is no definitive answer to this disturbing question.

In his discussion Douglas Porter uses the terminology of values to emphasize the normative dimension of diagnostic constructs from the outset. We don’t do science and then pragmatics. Our science has a normative dimension from our first moment on the proverbial bench. Joel Paris adds that we have become dazzled by a neuroscience that has thus far offered us minimal guidance, and that in the meantime we should continue adjusting our nosology in a practical way and adjust the DSM accordingly. Finally, Joseph Pierre ends with a provocative issue that again catches Frances’ attention. He argues that in the real-life world of clinical work we tend to treat the suffering that presents itself in our consulting rooms, with minimal regard for whether that suffering matches the thresholds of our categories; and he then argues that this diagnosis-creep will inexorably lead toward cosmetic enhancement. To be sure, he is taking us where many of us, Frances included, don’t want to go.

## Competing interests

MF is an external consultant to the NIMH Research Domain Criteria (RDoC) Project. NG has research grants from Pfizer and Sunovion, and is a research consultant for Sunovion. MS is a consultant for AstraZeneca, Merck, Novartis and Sunovion. Other authors report no competing interests.

## Authors’ contributions

JP(Phillips) wrote the general General Introduction and Conclusion, as well as the introductions to the individual conclusions. AF wrote the Responses to Commentaries. MC, JC, HD, MF, NG, GG, AH, WK, SL, EM, AM, JP(Paris), RP, HP, DP, CP, MS, TS, JW, SW, OW, PZ wrote the commentaries.
